# Loss of NOD2 in macrophages improves colitis and tumorigenesis in a lysozyme-dependent manner

**DOI:** 10.3389/fimmu.2023.1252979

**Published:** 2023-10-09

**Authors:** Camille Chauvin, Katarina Radulovic, Olivier Boulard, Myriam Delacre, Nadine Waldschmitt, Paul Régnier, Gauthier Legris, Clément Bouchez, Mohamed-Yassine Sleimi, Philip Rosenstiel, Guillaume Darrasse-Jèze, Mathias Chamaillard, Lionel F. Poulin

**Affiliations:** ^1^ Univ. Lille, Institut National de la Santé Et de la Recherche Médicale (Inserm), Centre de Recherche Hospitalier Universitaire (CHU) Lille, Institut Pasteur de Lille, U1019, Lille, France; ^2^ Institut national de la santé et de la recherche médicale (INSERM) U1138, Centre de Recherche des Cordeliers, Paris, France; ^3^ Unité de Recherche Clinique, Centre Hospitalier de Valenciennes, Valenciennes, France; ^4^ Univ. Lille, Inserm, U1003, Lille, France; ^5^ Chair of Nutrition and Immunology, School of Life Sciences, Technische Universität München, Freising-Weihenstephan, Germany; ^6^ Immunology-Immunopathology-Immunotherapy (i3) Laboratory, Institut national de la santé et de la recherche médicale (INSERM) UMR-S 959, Sorbonne Université, Paris, France; ^7^ Biotherapy Unit (CIC-BTi), Inflammation-Immunopathology-Biotherapy Department (DHU i2B), Groupe Hospitalier Pitié-Salpêtrière, Assistance Publique-Hôpitaux de Paris (AP-HP), Paris, France; ^8^ Institute of Clinical Molecular Biology, Christian Albrechts University and University Hospital Schleswig-Holstein, Kiel, Germany; ^9^ Université de Paris, Paris Descartes, Faculté de Médecine, Paris, France; ^10^ Université Paris Cité, Faculté de Médecine, Paris, France

**Keywords:** lysozyme, myeloid, colitis, cancer-associated colitis, NOD2

## Abstract

**Background:**

Crohn’s disease (CD) is a complex and poorly understood myeloid-mediated disorder. Genetic variants with loss of function in the *NOD2* gene confer an increased susceptibility to ileal CD. While Nod2 in myeloid cells may confer protection against T-cell mediated ileopathy, it remains unclear whether it may promote resolution of the inflamed colon. In this study, we evaluated the function of Nod2 in myeloid cells in a model of acute colitis and colitis-associated colon cancer (CAC).

**Methods:**

To ablate Nod2 specifically within the myeloid compartment, we generated *LysM^Cre/+^;Nod2^fl/fl^* mice. The role of NOD2 was studied in a setting of Dextran Sodium Sulfate (DSS)-induced colitis and in azoxymethane (AOM)/DSS model. Clinical parameters were quantified by colonoscopy, histological, flow cytometry, and qRT-PCR analysis.

**Results:**

Upon DSS colitis model, *LysM^Cre/+^;Nod2^fl/fl^* mice lost less weight than control littermates and had less severe damage to the colonic epithelium. In the AOM/DSS model, endoscopic monitoring of tumor progression revealed a lowered number of adenomas within the colon of *LysM^Cre/+^;Nod2^fl/fl^* mice, associated with less expression of *Tgfb*. Mechanistically, lysozyme M was required for the improved disease severity in mice with a defect of NOD2 in myeloid cells.

**Conclusion:**

Our results indicate that loss of Nod2 signaling in myeloid cells aids in the tissue repair of the inflamed large intestine through lysozyme secretion by myeloid cells. These results may pave the way to design new therapeutics to limit the inflammatory and tumorigenic functions of NOD2.

## Highlights

**What is already known?**

- CD patients with NOD2 mutations have a lower incidence of colitis than ileitis.
- Mice deficient for Nod2 are more susceptible to colitis and colitis-associated colon cancer
- Nod2 expression in phagocytes can decrease mucosal damage and immune response in small intestine enteropathy.
- Lysozyme P is normally secreted by Paneth cells in the small intestine (and ascending colon in humans) in a NOD2-dependent manner, and the presence of lysozyme-producing Paneth cells in the distal colon and rectum has been associated with IBD in humans.
- While NOD2 can control the secretion of lysozyme P, lysozyme-P deficiency has been shown to decrease NOD2 signaling and to alter the microbiota resulting in the protection of the mice from colitis.

**What is new here?**

- Loss of Nod2 signaling in myeloid cells contributes to tissue repair in the inflamed large intestine and decreases adenomas through a lysozyme-dependent mechanism.

**How can this study help patient care?**

- This study might contribute to the development of novel therapeutic strategies to limit the inflammatory and tumorigenic functions of NOD2.

**Lay summary (40 words)**

Loss of Nod2 signaling in myeloid cells contributes to tissue repair in the inflamed large intestine through lysozyme secretion by myeloid cells, which may explain the lower incidence of colitis in Crohn’s disease patients with NOD2 mutations.

## Introduction

Crohn’s disease (CD) is a complex and poorly understood myeloid-mediated disorder. The prevalence of patients suffering from CD has been increasing remarkably over the last decades (1). CD lesions are characterized by an overt accumulation of inflammatory macrophages and neutrophils in any part of the gastrointestinal tract (2). An increased number of inflammatory macrophages was observed within the intestinal mucosa of CD patients at the expense of their pro-resolving counterparts (3). Those data were supported by a recent single-cell analysis of inflamed tissues from CD, which revealed the presence of a discrete subset of pathogenic macrophages within the diseased intestine of CD patients who fail to respond to anti-TNF therapy (4). In association with the fact that inflammation may play a driving role at all stages of tumorigenesis (5), a substantial portion of CD patients with long-lasting colitis are at elevated risk of developing colitis-associated colorectal cancer (CAC) (6).

CD is believed to have a pathogenesis caused by a complex interaction with the gut microbiota in genetically susceptible individuals (7, 8). Genetic variants in the *NOD2* gene confer an increased susceptibility to CD, likely due to the loss of the Nucleotide-binding Oligomerization Domain (NOD)-like receptor (NOD2) function. While patients who carry NOD2 risk alleles are at greater risk of developing stricturing disease, others and we have indicated that *Nod2*-deficient mice are highly susceptible to DSS-mediated colitis and CAC and that the dysbiosis in NOD2-deficient mice could sensitize colonic mucosa to cancer (9–11). The global protective role of NOD2 in gut inflammation and colitis has been evaluated in various studies. NOD2 deletion ameliorated colitis severity in IL-10^-/-^ mice and this effect was dependent on the gut microbiota composition (12). The role of NOD2 has been demonstrated in an ileitis-prone mouse model, where NOD2 ablation weakened the severity of spontaneous intestinal disease without affecting the microbial composition (13). However, the role of Nod2 in myeloid cells in a model of acute colitis and colitis-associated colon cancer (CAC) has not been clearly elucidated. Primarily expressed in myeloid cells, NOD2 is a cytosolic sensor of bacterial muramyl dipeptide (MDP), a product of the hydrolysis of the peptidoglycan (PG) present on either gram-negative or positive bacterial wall (14). In hematopoietic cells, Nod2 has been shown to regulate gut homeostasis and permeability and to be involved in the sensibility to inflammation of the gut mucosa (15). NOD2 activation leads to the production of inflammatory cytokines through NF-kB and MAPK (14). It is established that the cytokine response to lipopolysaccharide (LPS) by macrophages with intact NOD2 signaling is lowered when primed by MDP (16). By contrast, MDP-stimulated macrophages from patients bearing CD-associated NOD2 mutations failed to decrease the responsiveness to LPS (16). Accordingly, a specific lack of control of NF-kB activation in myeloid cells results also in a higher susceptibility to DSS-induced colitis (17).

It is thereby tempting to speculate that the protective function of NOD2 within macrophages is by limiting the production of inflammatory effectors in response to bacterial-derived molecules. Nod2 expression in phagocytes can decrease mucosal damage and immune response in a preclinical model of T cell-induced small intestine enteropathy triggered by acute T cell activation following anti-CD3 antibody injection (18). However, it remains unclear why the involvement of the transverse colon, left colon, or rectum was significantly less common among CD patients bearing NOD2 mutations (19). The understanding of the cellular and molecular functions of NOD2 is of essential importance for the development of novel therapeutic strategies to treat and prevent this disease. In the present study, the use of the LysMcre knock-in/knock-out mice led us to unveil that specific ablation of Nod2 expression in myeloid cells renders mice more resistant to colitis and CAC in a lysozyme-dependent manner.

## Results

### Lack of Nod2 signaling in myeloid cells does not affect the composition of intestinal phagocytes before and after acute colitis

**Figure 1:**
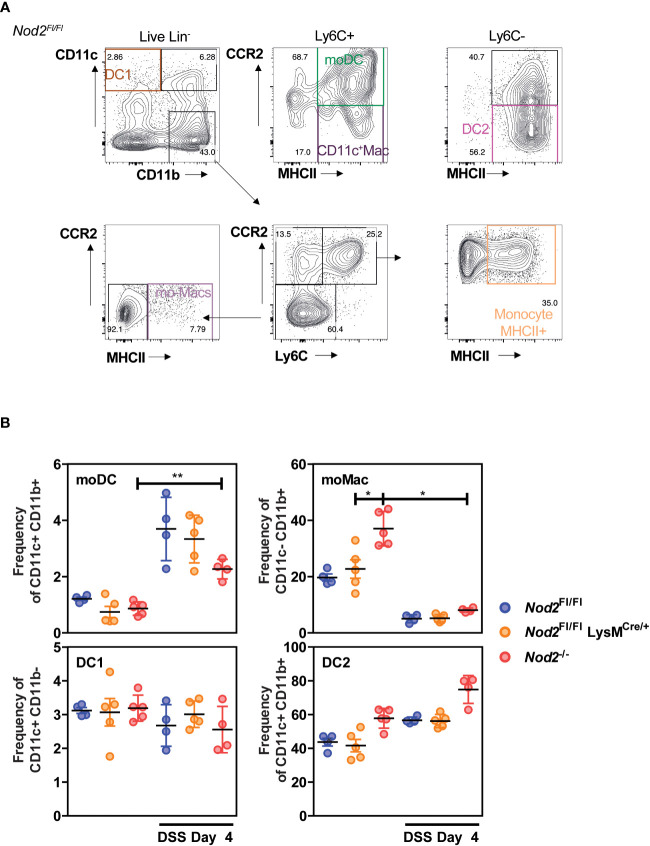
Colonic phagocyte composition was not affected by the lack of Nod2 in myeloid cells. **(A, B)** *LysM^Cre/+^;Nod2^fl/fl^* mice (orange) were compared to control flox mice (blue) and *Nod2^-/-^* (red) before and after induction of DSS-mediated colitis. Gating strategy **(A)** to determine the frequency **(B)** of conventional DC1 (CD11c^+^CD11b^-^), of mo-DCs (CD11c^+^CD11b^+^Ly6C^+^CCR2^+^MHCII^+^), of CD11c^+^ Macs (CD11c^+^CD11b^+^Ly6C^+^CCR2^-^MHCII^+^), of conventional DC2 (CD11c^+^CD11b^+^Ly6C^-^CCR2^-^MHCII^+^), and the frequencies of mo-Macs (CD11c^-^CD11b^+^Ly6C^-^CCR2^-^MHCII^+^), and Monocyte-MHCII^+^ (CD11c^-^CD11b^+^Ly6C^+^CCR2^+^MHCII^+^), based on their CD11c, CD11b, CCR2, Ly6C, and MHCII levels, after exclusion of Lineage and doublet cells. There were 4-5 mice per group. Bars indicate the mean ± SEM. The gating strategy in A was performed on NOD2^fl/fl^ mice at day 4 of DSS, the same strategy was applied to the other genotypes and experimental conditions. Statistical significance was assessed by a non-parametric Mann-Whitney test, or by two-way ANOVA, Bonferroni’s multiple comparisons test **(B)**. *P<0.05; **P<0.01.

We first assessed whether Nod2 expression in myeloid cells, including monocyte-derived phagocytes such as monocyte-derived dendritic cells (mo-DCs) and macrophages (mo-Macs) (20), may modulate the myeloid content at steady state and upon acute colitis. To this end, Nod2^fl/fl^ mice were crossed with mice expressing the recombinant Cre in myeloid cells, *LysMCre* (21). Lysozyme expression was found in bone-marrow-derived macrophages (22) and in macrophage aggregates in the lamina propria in 7 out of 20 CD patients (23). As expected, *Nod2* expression was lowered in the peritoneal macrophages (but not on CD4 T cells) of *LysM^Cre/+^;Nod2^fl/fl^* mice, which are herein referred to as Nod2^▵Lyz2^, compared to their Nod2^fl/fl^ littermates (**Supplementary Figure 1A**). In agreement, *Nod2* expression was lowered in the M-CSF culture of bone marrow cells from Nod2^▵Lyz2^ cells, as compared with Nod2^fl/fl^ cells (**Supplementary Figure 1A**). Live single-cell suspensions were next isolated from the colon of Nod2^ΔLyz2^ mice and littermate controls before being analyzed by multiparametric flow cytometry (**Figure 1A**). After the exclusion of dead cells, doublets and lineage-positive cells, a CD11c-CD11b dot plot was subdivided according to the subset of hematopoietic cells expressing CCR2, Ly6C and then subdivided according to the absence or presence of major histocompatibility complex class II (MHCII) on their cell surface to identify mo-Macs (CD11c^-^ CD11b^+^ CCR2^-^ Ly6C^-^ MHCII^+^) and activated monocytes (Monocyte MHCII^+^) (CD11c^-^ CD11b^+^ CCR2^+^ Ly6C^+^ MHCII^+^) (**Figure 1B**). Given that Nod2 has been reported to shape CD103^+^ DC recruitment in the gut lamina propria (24), we first reasoned that loss of Nod2 signaling may have altered trafficking or development of discrete subsets of conventional dendritic cells that do not express Ly6C and are largely dependent on GM-CSF, also known as colony-stimulating factor 2 (Csf2). To our surprise, only minor fluctuations were detected at steady-state and under DSS in the frequency of cDC1 and cDC2, co-expressing or not CD11b respectively. As observed for cDCs, the frequencies of mo-DCs co-expressing the surface molecules CD11b, CD11c, Ly6C, CCR2, and MHCII (25), did not differ between Nod2^ΔLyz2^ and littermate controls at steady-state, but significantly increased upon inflammation. The number of CD11c-expressing macrophages was too low to capture potential differences with our experimental setting conclusively. The heterogeneity within the CD11b^+^CD11c^-^ cells from the colon of Nod2^ΔLyz2^ mice and littermate controls were next studied in further detail. Although no difference in the proportions of mo-Macs and activated monocytes (data not shown) was observed between Nod2^ΔLyz2^ mice and littermate controls, *Nod2*-deficient mice contain a higher frequency of mo-Macs at steady-state which may be caused by their dysbiosis (**Figure 1B**). Upon acute exposure to dextran sodium sulfate (DSS), the myeloid content of *LysM^Cre/+^;Nod2^fl/fl^* mice do not show significant fluctuations with our antibody combinations when compared with their littermates. Collectively, flow cytometry analysis of mononuclear phagocytes from the large intestine of littermates revealed that acquired loss of Nod2 in myeloid cells was not sufficient to trigger significant fluctuations of discrete phagocytic subsets at baseline.

### Loss of Nod2 signaling in myeloid cells improves intestinal repair after acute colitis

**Figure 2:**
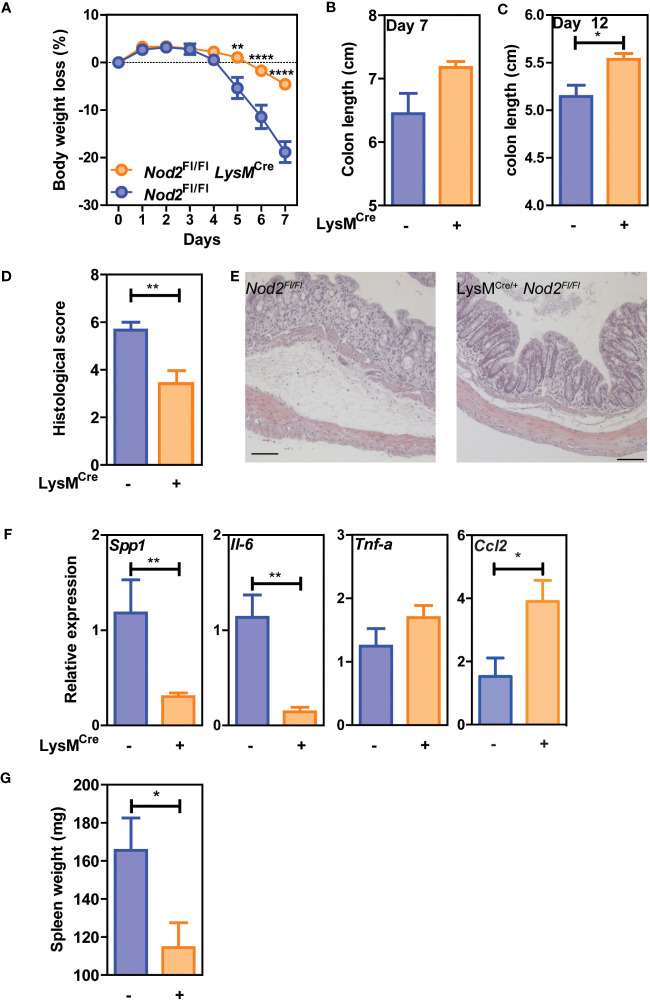
Nod2 deficiency in mature mononuclear phagocytes is protective in colitis. *LysM^Cre/+^;Nod2^fl/fl^* were compared to control flox mice after induction of DSS-mediated colitis. The weight loss was monitored during DSS-mediated colitis **(A)**, as well as the colon length at day 7 **(B)** and day 12 **(C)** and the spleen weight **(G)**. Histological sections in H&E were done to confirm tissue damages at day 7 **(D, E)**. **(F)** RT-qPCRs on colonic tissues were done to measure *Il-6*, *Tnf-a*, *Spp1*, and *Ccl2* at day 7 after DSS induction (5-6 mice per group). Representative of 2 independent experiments. Bars indicate the mean ± SEM. Statistical significance was assessed by a non-parametric Mann-Whitney test, or by two-way ANOVA, Bonferroni’s multiple comparisons test. *P<0.05; **P<0.01; ****P<0.0001.

We then assessed whether Nod2 expression in macrophages was required for proper healing following colon tissue damage induced by DSS. Nod2^ΔLyz2^ and littermate controls were exposed to a 5-day cycle of DSS followed by a recovery phase of 2 days. Daily monitoring of body weight and signs of rectal bleeding and diarrhea revealed that Nod2^ΔLyz2^ mice lost less body weight than their similarly challenged control littermates as early as five days of DSS exposure (**Figure 2A**). By 7 days, colon shortening was not significantly different between the groups (**Figure 2B**). However, other groups of mice followed until 12 days after the beginning of DSS treatment, exhibited a decrease of colon shortening in Nod2ΔLyzM mice in the recovery phase of 7 days after the end of DSS (**Figure 2C**), which is consistent with a less damaged colon based on histological sections at day 7 (**Figures 2D, E**), and suggesting that mice with NOD2 deletion in macrophages are less susceptible to acute colitis. We also observed a decrease in spleen weight compared with control littermates at 7 days (**Figure 2G**). While *Ccl2* was upregulated in the colonic tissues of Nod2^ΔLyz2^ mice after 5 days of DSS exposure and 2 days of recovery suggesting the rapid recruitment of monocyte-derived cells to the inflammatory site, inflammatory markers such as *Spp1 and Il-6* and were less expressed compared with control animals implying a reduced colonic inflammation and macrophage activation when Nod2 was depleted in macrophages (**Figure 2F** and data not shown). However, we did not notice any change in the transcript levels of *Tnf-α*, *Ido1*, *Cxcl9*, and *Cxcl10*, which are markers of inflammation or intestinal recruitment measured in the whole colonic tissue (**Figure 2G** and data not shown) (26). Taken together, our data suggest that bacterial sensing by intestinal myeloid cells via NOD2 promotes inflammation during acute colitis and impairs the intestinal repair phase after acute colitis. Alternatively, it is also possible that a yet-to-be-identified compensatory mechanism may somehow render Nod2^ΔLyz2^ more resistant to acute DSS-induced injury.

### Colitis-associated carcinogenesis is reduced in Nod2^ΔLyz2^ mice

**Figure 3:**
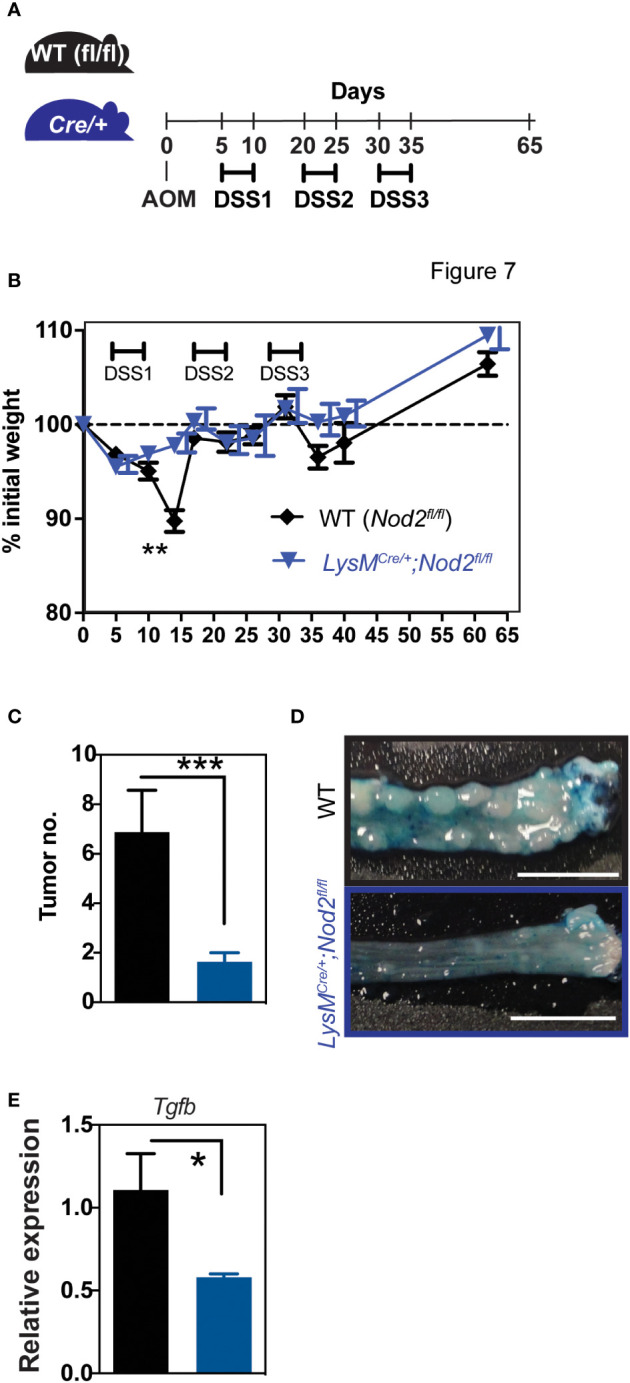
Nod2 deficiency in mature mononuclear phagocytes is protective in colorectal cancer. **(A)** *LysM^Cre/+^;Nod2^fl/fl^* mice were compared to control flox mice after induction of colorectal cancer by AOM/DSS. The weight loss was monitored over time **(B)** and the development of colonic tumors was confirmed at the sacrifice **(C, D)**. The relative expression of *Tgfb* was quantified by RT-qPCR on tumoral tissue **(E)**. *LysM^Cre/+^;Nod2^fl/fl^* (n=7) and control flox mice (n=10). Bars indicate the mean ± SEM. Statistical significance was assessed by a non-parametric Mann-Whitney test or by two-way ANOVA, Bonferroni’s multiple comparisons test. *P<0.05; **P<0.01; ***P<0.001 were considered statistically significant.

Given the paradigm that the myeloid compartment may contribute to colorectal cancer (CRC) progression through its inflammatory and immunosuppressive properties (27), we next asked whether Nod2 signaling in myeloid cells could intrinsically regulate tumor progression in the colitis-associated carcinogenesis model. Thus, Nod2^ΔLyz2^ and their control littermates were treated with the AOM-DSS protocol, in which after injection of the AOM carcinogen, 3 cycles of inflammation were performed by adding 2% DSS in drinking water for 5 days (**Figure 3A**). As observed with DSS-induced colitis, we observed protection to weight loss in Nod2^ΔLyz2^ during the first cycle of DSS in the AOM-DSS protocol (**Figure 3B**). Accordingly, endoscopic monitoring of tumor progression revealed a decrease in adenomas in Nod2^ΔLyz2^ mice (**Figures 3C, D**; **Supplementary Figure 2**). To better understand the mechanisms leading to protection in Nod2^ΔLyz2^, the expression of *Ccl2, Cxcl1* and genes related to cell functions, such as *Tgfb*, *Il10*, *iNos*, *Areg*, *Cxcl13*, and *Ptgs2*, was quantified by RT-qPCR on tumoral tissue at the end of the AOM/DSS protocol (**Figure 3E**, **Supplementary Figure 3** and data not shown). The expression of *Ccl2 and Cxcl1* were not significantly different between Nod2^ΔLyz2^ and their littermate controls when measured at the end of the experiment (**Supplementary Figure 3**). Nevertheless, myeloid cells could have been recruited earlier, during the first or second cycle of colitis as suggested early with the acute colitis protocol. In addition, tumors from Nod2^ΔLyz2^ were expressing less *Tgfb* than the controls (**Figure 3E**), which corresponds to a less activated state generally associated with cancer evasion and metastasis. Indeed, an imbalance of TGF-β expression has been observed in the intestines of IBD patients and in mouse models (28) and high TGF-β expression has been found in strictures of CD patients (29). Tumor metastasis inhibition has been described upon specific genetic deletion of *Tgfbr2* in myeloid cells (30) suggesting the role of myeloid-produced TGF-β in cancer progression and possibly explaining the reduced tumor numbers in Nod2^ΔLyzM^ mice. Overall, our data indicate that loss of Nod2 signaling in lysozyme-expressing myeloid cells lowers carcinogenesis in the context of colitis.

### NOD2 links a gene signature of monocyte-derived phagocytes with prognosis in colorectal cancer

**Figure 4:**
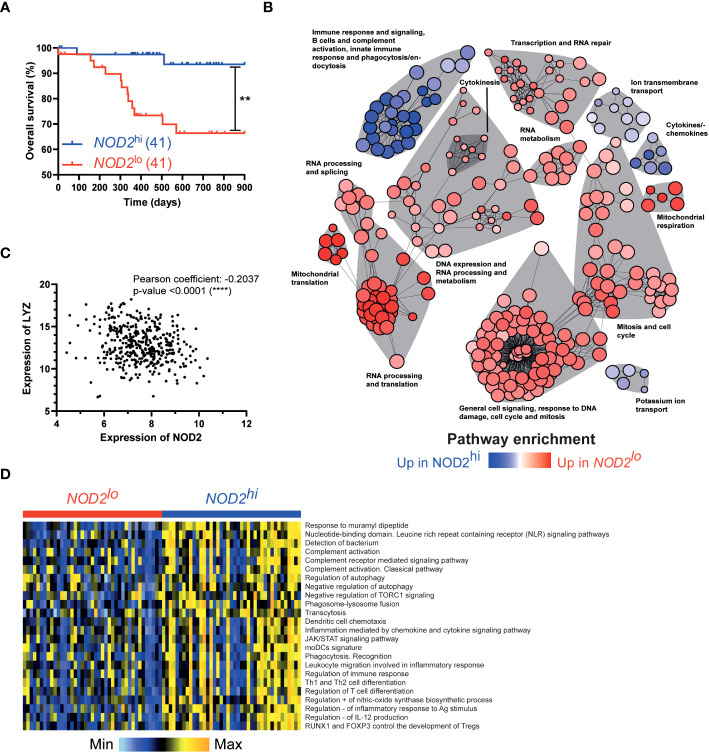
NOD2 links a gene signature of phagocytes with prognosis in colorectal cancer. Patients were separated into 2 groups according to their expression of NOD2: *NOD2^lo^* (red; 41 patients) and *NOD2^hi^* (blue; 41 patients) (data publicly available from TCGA). Survival curves were compared using a log-rank (Mantel-Cox) test. **,P<0.01. **(B)** Network analysis of significantly dysregulated pathways between *NOD2^lo^* (red) and *NOD2^hi^* (blue) groups. **(C)** Correlation between *LYZ* and *NOD2* expression. **(D)** Heatmap of selected pathways to compare *NOD2^lo^* and *NOD2^hi^* groups.

To assess the potential prognostic value of NOD2 in colorectal cancer, we assessed the transcriptome of 402 colorectal tumors from the TCGA-COAD cancer project that was available in the data repository of the Cancer Genome Atlas. Before any comparison to clinical outcome, we first discriminated the patients according to their *NOD2* gene expression levels, using 10% (n = 41 patients) as the lower and upper threshold. In this setting, the 10% most-expressing patients were defined as NOD2^hi^ whereas the 10% least-expressing patients were defined as NOD2^lo^. Interestingly, while NOD2 has been shown to be significantly highly expressed in CRC patients as compared to healthy controls (31), we observed a strongly increased overall survival as well as median survival time in NOD2^hi^ patients as compared to NOD2^lo^ patients (**Figure 4A**). These data highlight the global role of bacterial sensing by NOD2 in inducing a beneficial immune response to combat cancer progression. On the other hand, reduced expression of NOD2 is associated with a worse prognosis in CRC patients. Then, the transcriptomic data from NOD2^hi^ and NOD2^lo^ tumors were further analyzed with *GSVA* and *limma* R packages using more than 5,000 gene sets coming from several well-known databases. This led us to identify a total of 1,778 pathways that were differentially enriched between the two settings (adjusted p-value < 0.05) (**Supplementary Table 1**). To get further insights, gene sets network analysis and representation were performed using *igraph* R package based on the previously computed pathways enrichments between NOD2^lo^ and NOD2^hi^ TCGA-COAD patients. Global pathway analysis uncovered several functional categories that are related to immune system processes among tumor specimens with the highest levels of NOD2 expression (**Figure 4B**). As expected, the pathway of immune response and signaling, B cells, and complement activation, innate immune response and phagocytosis/endocytosis, as well as the cytokines/chemokines pathway were upregulated in NOD2^hi^ patients, but also the ion transmembrane transport pathway and the potassium ion transport pathway. These pathways suggest a hyper-activated status in NOD2^hi^ patients, in line with the higher activation and immune response found in Crohn’s disease patients without NOD2 mutations, exhibiting increased cytokine receptors and decreased microRNA expression (32). Complement activation can trigger powerful phagocytic chemotaxis in the event of insoluble PG accumulation due to reduced digestion of this extracellular compound by lysozymes (33). Accordingly, LYZ expression correlated negatively and significantly with NOD2 expression (Pearson = -0.2037 and p-value <0.0001, and Spearman = -0.1954 and p-value <0.0001) (**Figure 4C**). LYZ was associated with the Gene Ontology pathway GO:0042742 “Defense Response To Bacterium”, which we found upregulated in NOD2^hi^ patients. Additionally, different pathways were associated in NOD2^hi^ patients to an immunosuppressive environment with for instance regulatory T cells development, positive regulation of nitric-oxide synthase biosynthetic process and negative regulation of IL-12 production (**Figure 4D**). Interestingly, several pathways involved in RNA repair, processing, and metabolism were overexpressed in NOD2^lo^ patients. In addition, the pathways of translation and mitochondrial respiration were overexpressed in NOD2^lo^ patients. The latter is associated with a protective anti-metastatic program formed by macrophages and associated with higher levels of mitochondrial reactive oxygen species (mtROS) (34). These data indicate different inflammatory pathways between NOD2^hi^ and NOD2^lo^ patients, leading to better survival of NOD2^hi^ individuals. However, these data also suggest that we cannot exclude a detrimental role for NOD2 in myeloid cells.

### LPS pretreatment increases the responsiveness to MDP of terminally differentiated macrophages

To further investigate the role of NOD2 signaling on the function of terminally differentiated macrophages, Nod2^+/+^ THP1 or Nod2^-/-^ THP1 cells were cultivated with Phorbol 12-myristate 13-acetate (PMA) for 2 days followed by 3 days of rest for generating macrophages (PMA-Mac), as previously described (35). Inflammatory cytokines secretion was next measured upon 24h treatment of PMA-Mac with LPS followed by 24h treatment with MDP or only upon MDP treatment. MDP is known to synergistically promote the proinflammatory cytokine expression induced by LPS in monocyte cell culture (36). In agreement with *in vivo* data using Nod2^ΔLyz2^, greater responsiveness to subsequent MDP stimulation was noted when PMA-Mac were first primed with LPS as determined by TNF-α quantification (**Supplementary Figure 4**). These data highlight the pro-inflammatory state of NOD2-competent macrophages, which is weakened when macrophages are deficient in NOD2. These results suggest that terminally differentiated macrophages may undergo a phenotypic switch toward a proinflammatory function upon activation of Nod2 signaling. Another explanation of our observations could be that myeloid-specific Nod2 signaling might increase the suppressive ability of these cells as demonstrated for Nod1 in peritoneal phagocytes (37). We tested whether MDP-treated peritoneal phagocytes had increased immunosuppressive potential in the same type of *in vitro* T-cell activation assay in the presence of myeloid cells. We first set up our suppression assay by using myeloid cells from FK-565-treated mice, a Nod1 agonist used as a positive control. As expected, myeloid cells obtained from mice injected intraperitoneally with the Nod1 agonist suppressed CD8 T cell proliferation (data not shown). Interestingly, we observed an absence of T-cell proliferation as compared to T cells only when CD8^+^ T cells were co-cultured with peritoneal phagocytes pre-treated *in vivo* for 24h with MDP (Myeloid^MDP^) (**Supplementary Figure 5**). In contrast, we measured T-cell proliferation in the presence of peritoneal myeloid cells isolated from control mice (Myeloid), suggesting that phagocytes from Myeloid^MDP^ mice exhibit a suppressive effect on T-cell activation. These results suggest that Nod2 signaling on myeloid cells may favor inflammatory and suppressive responses.

### The improvement of Nod2^ΔLyzM^ mice in a model of acute colitis and CAC relies on the compensatory expression of lysozyme M

Classical monocytes have the ability to be converted into non-classical cells in a NOD2-dependent manner (38). As Nod2 stimulation has been shown to increase lysozyme secretion by Paneth cells within the crypt (39) and NOD2 mutations in CD patients have been linked to a dysregulation of lysozyme-containing granules (40), we could hypothesize that NOD2 might influence LyzM expression in myeloid cells. We therefore analyzed previously published RNAseq data from classical Ly6C^lo^ mouse monocytes treated or not with MDP (GEO accession number GSE101496) for *Lyz2* expression. *Lyz2* expression is significantly reduced by 2-fold in these cells after MDP treatment (p-value 0.0247). In addition, we used a published RNA-seq dataset from an exploratory cohort deposited in the GEO database (GSE69446) (41) and observed a similar trend in terms of *LYZ* expression, which is not observed in patients carrying a NOD2 frameshift (32). In agreement, we have previously observed that transferring flora from Nod2-deficient mice to control mice increases the intestinal expression of the *Lyz2* gene (11). Thus, the absence of Nod2 in phagocytes could up-regulate lysozyme expression in phagocytes.

Lysozyme-expressing CD102^+^ (ICAM2)^+^ macrophages are recruited in a CCR2- and antibiotic-independent manner to the colon in DSS-induced colitis and have been proposed to promote intestinal repair (42). To determine if Nod2 plays an intrinsic role in the extravasation of these lysozyme-expressing CD102^+^ peritoneal macrophages to the colon in DSS-mediated colitis, we made Nod2-deficient competitive bone marrow (BM) chimeras (as described recently (41), **Supplementary Figure 6A**). We observed a decreased abundance of Nod2-deficient CD102^+^ peritoneal macrophages (CD45.2) when compared with wild-type controls (CD45.1) (**Supplementary Figure 6B**). Thus, the lack of Nod2 in phagocytes could also control the migration of lysozyme-expressing macrophages to favor intestinal repair.

**Figure 5:**
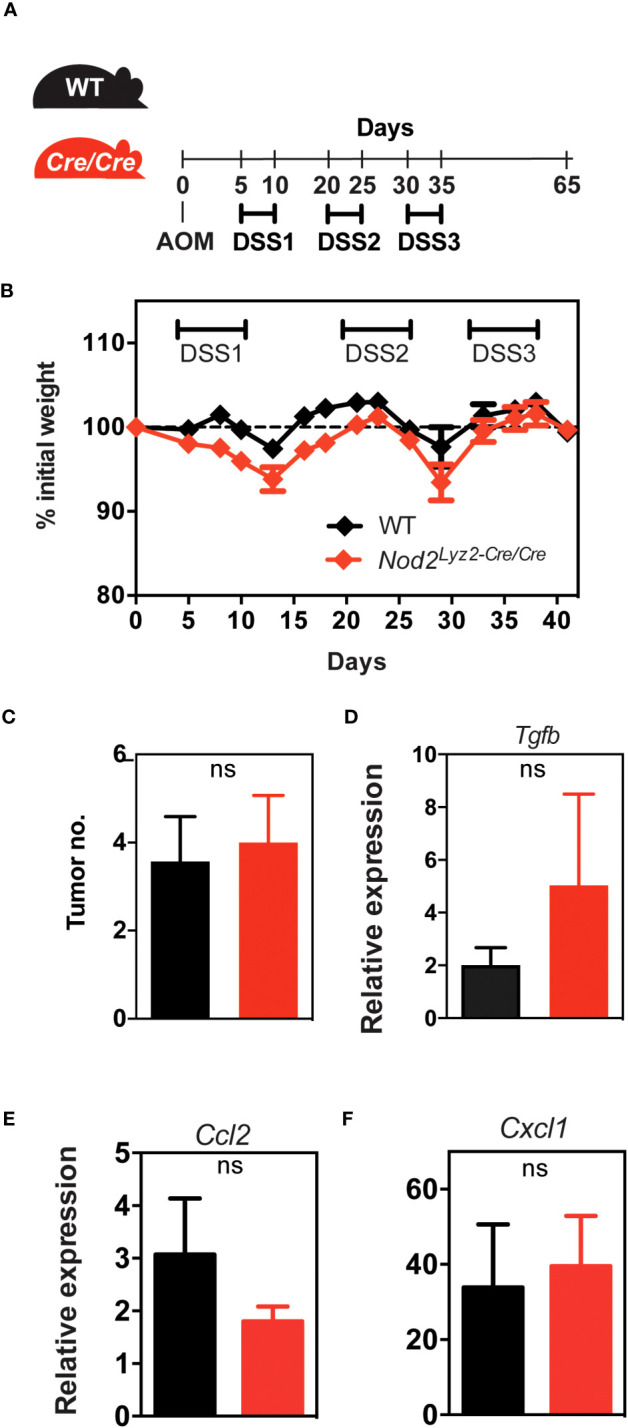
Lysozyme M controls the colorectal protective effect in the absence of Nod2 in myeloid cells.**(A)** *LysM^Cre/Cre^;Nod2^fl/fl^* mice were compared to control WT mice after induction of colorectal cancer by AOM/DSS. **(B, C)** The body weight loss was monitored over time and the development of colonic tumors was assessed as described in **Figure 3**. The relative expression of *Tgfb* **(D)**, *Ccl2* **(E)**, and *Cxcl1* **(F)** was quantified by RT-qPCR on tumoral tissue. Representative of 2 independent experiments (n>5 mice per group). Bars indicate the mean ± SEM. Statistical significance was assessed by a non-parametric Mann-Whitney test. ns, non-significant.

We then investigated whether the protection, in terms of weight loss in DSS-induced colitis and AOM/DSS-induced CAC, observed in *LysM^Cre/+^;Nod2^fl/fl^* mice depended on lysozyme M expression. We generated mice that are deficient for both Nod2 and lysozyme M in myeloid cells (herein referred to as *LysM^Cre/Cre^;Nod2^fl/fl^* mice) by taking advantage of the fact that the insertion of the gene encoding Cre rendered the Lyz2 gene nonfunctional. By contrast to what was observed in *LysM^Cre/+^;Nod2^fl/fl^* mice, *LysM^Cre/Cre^;Nod2^fl/fl^* mice were not protected in terms of weight loss in the DSS-induced colitis model (data not shown). Similarly, in the AOM/DSS-induced CAC model (**Figure 5A**), *LysM^Cre/Cre^;Nod2^fl/fl^* mice were as susceptible to body weight loss as WT mice (**Figure 5B**). After determining the day of autopsy using the endoscope, the number of tumors and the colon weight were not significantly different between the *LysM^Cre/Cre^;Nod2^fl/fl^* mice and controls (**Figure 5C**; data not shown). The tumor microenvironment in each genotype was next studied by histological analysis with hematoxylin-eosin staining and no differences were observed between WT and mice lacking specifically Nod2 and lysozyme M in myeloid cells (data not shown). In addition, we could observe that the difference in the transcript level of *Tgfb* expression was lost between *LysM^Cre/+^;Nod2^fl/fl^* mice and their control littermates (**Figure 5D**). We did not measure significant differences in the transcript level of Ccl2 and Cxcl1 expression in these mice (**Figures 5E, F**).

In this model, our results indicated that the loss of LyzM specifically in myeloid cells did not confer the anti-inflammatory response observed in the *LysM^Cre/+^;Nod2^fl/fl^* mice and that the anti-tumor protection is dependent on the expression of the anti-bacterial molecule, lysozyme M.

## Discussion

It is well established that Nod2 primarily encodes a sensor of lysozyme-digested PG in myeloid cells, as opposed to Nod1, which is widely produced by a variety of cell types such as lymphocytes. Our results herein provide evidence that loss of Nod2 signaling in either macrophages or neutrophils mediates protection against intestinal inflammation and tumorigenesis. Specifically, LysM^Cre/+^;Nod2^fl/fl^ mice showed less severity in DSS-induced colitis as evidenced by a decreased production of inflammatory markers when compared to that in their wild-type littermates. Accordingly, tumor growth was reduced in LysM^Cre/+^;Nod2^fl/fl^ mice in a model of CAC. Mechanistically, the attenuated symptoms of LysM^Cre/+^;Nod2^fl/fl^ mice relied on the expression of lysozyme M that is able to promote monocyte recruitment in a Nod-dependent manner (43). Therefore, supplementation with lysozyme derived from hen egg attenuated the severity of colitis induced by DSS and improved nutrient absorption (44). Lysozyme is a hydrolytic enzyme that is secreted in a variety of fluids for promoting host defense. It hydrolyzes the glycosidic bonds between N-acetylmuramic acid and N-acetylglucosamine. Consequently, products of the digestion of PG by lysozyme M are able to promote the release of muropeptides that potently activate Nod2 signaling (43), as it was also observed when lysozyme P liberates Nod1 agonists (45). Of note, the secretion of lysozyme P by Paneth cells is enhanced in the small intestine of mice with a defect in lysozyme M, suggesting the possibility of a proinflammatory role of Nod1 signaling in LysM^Cre/Cre^;Nod2^fl/fl^ mice. This enhanced secretion may be due to a higher Nod2 activity which has been shown to regulate lysozyme P sorting and secretion by Paneth cells within the crypt through the Receptor-interacting serine/threonine-protein kinase 2 (Rip2) pathway (39). On the other hand, other factors may explain our findings as in human Paneth cells, lysozyme, like other antimicrobial peptides, exhibits antibacterial and antimicrobial activities that may be impaired in CD patients (15).

Many aspects of this study warrant further explanation and comments. The absence of significant differences in the composition of the gut microbiota of *LysM^Cre/+^;Nod2^fl/fl^* mice and their wild-type littermates (data not shown) led us to favor the possibility of a greater Nod signaling in other cell types than myeloid cells independently of bacterial community structure. DSS administration in WT mice significantly increases *Lyz1* and *Lyz2* expression in the colon (45). Lyz1, which encodes lysozyme P, is expressed and secreted principally by colonic Paneth cells; on the other hand, Lyz2, which encodes lysozyme M, is produced by phagocytes. *Lyz1^-/-^* mice are protected during DSS colitis and this protection is associated with changes in the microbiota composition (45). In agreement with our study, no difference was detected in the composition of the microbiota of mice deficient for Nod2 in the hematopoietic compartment (46). An additional question was whether our results could be transposed to other preclinical models of colitis. By contrast to what was herein observed with the acute model of colitis induced by DSS, higher numbers of epithelial apoptotic bodies and increased expression of TNF-α and IL-22 were observed in NOD2^▵Lyz2^ mice in a T cell-mediated model of ileopathy (18). The differential effect of NOD2 in a model of colitis and ileitis may explain why the involvement of the transverse colon, left colon, or rectum was significantly less common among CD patients bearing NOD2 mutations (19). As depicted for type-I IFNs which have opposing effects during the emergence and recovery phases of colitis, it would be of interest to study if our conclusions may apply also to the recovery phases of colitis (47). One potential mechanism to explain the Lyz2 dependency of our data is that Nod2 could control Lyz2 expression.

In Paneth cells, NOD2 is stimulated by PG hydrolysis, leading to activation of the LRRK2-RIPK2-RAB2A pathway, which triggers lysozyme sorting in the crypt (48) (49). However, lysozyme P secretion and function are impaired when NOD2 is dysfunctional. Upon bacterial entry in the cell and PG sensing, NOD2 also recruits ATG16L1 to the plasma membrane to induce autophagy. NOD2-induced autophagy has been described as Rip2-dependent in human DCs and leads to an enhanced anti-bacterial response (50). The link between NOD2, lysozyme M, and autophagy is still unclear. However, it has been shown that, during Salmonella typhimurium infection, Paneth cells can secrete lysozyme through an alternative autophagy-based pathway to protect the classical ER-Golgi pathway (51). Furthermore, ATG16L1T300A mutated mice show reduced lysozyme P secretion and bacterial clearance upon *S. typhimurium*, suggesting the control of lysozyme P secretion via the autophagy pathway (51). Also, Nod1 reprograms peritoneal myeloid cells through autophagy and arginase 1 induction, and Nod1^ΔLyz2^ mice are less susceptible to tumorigenesis (37). Further research is needed to decipher these links for lysozyme M in phagocytes in the absence or presence of a functional NOD2 signaling pathway.

The lysozyme in phagocytes may participate by various mechanisms in the reduction of inflammation and colorectal cancer in the absence of Nod2-mediated signaling. Lysozyme-mediated bacterial degradation in phagocytes is linked to the recruitment and activation of macrophages and neutrophils (52) (53). These phagocytes can promote the pro-inflammatory effect of Lysozyme M, which is associated with bacterial wall hydrolysis, pathogen-associated molecular pattern (PAMP) release, and direct or indirect NLR/TLR activation (33, 54). This pro-inflammatory effect is associated with an increase in neutrophil functions and a strong cytokine response such as IL-6 and TNF-α. Furthermore, it has been proposed that the absence of NOD2 results in a macrophage-fibroblast pathogenic program, in which STAT3 is suggested to sustain pro-inflammatory cytokines and pathogenic T cell activation for example (55). In this study, the gp130 family of genes (IL6, IL11, and OSM) and IL6ST (encoding gp130) have been shown to be upregulated in intestinal cells from patients with *NOD2* loss-of-function alleles (55). While further research is needed to explore the regulation of the gp130 pathway and IL6R, IL11R, OSMR, and LIFR which dimerize with gp130, by lysozyme M in phagocytes, we did not observe an increase of the inflammation in the absence of Nod2 in phagocytes. Additionally, gp130/IL6ST is not correlated to any pathways shown in **Figure 4**. By themselves alone, neither expression of LYZ nor IL6ST is associated significantly with patients’ survival in this database. Other parameters may influence the protective response observed such as the susceptibility of PG to lysozyme disruption, and the amount and composition of the factors released as a consequence. The better survival of NOD2hi COAD patients compared to NOD2lo patients confirms the importance of NOD2 signaling in the immune response against tumor, but may appear at odds with the lower carcinogenesis observed in mice with Nod2 deficiency in lysozyme-expressing myeloid cells. This discrepancy suggests that the consequence of NOD2 signaling varies depending on which kind of cells express it. This also suggests that the location (tumor biopsy in **Figure 4** vs global NOD2 deficiency in mice) and timing of NOD2 signaling (NOD2 expression in established tumor vs NOD2 deficiency initiated by lysozyme expression) may have very different consequences on the immune response / tolerance balance.

Lysozyme-mediated degradation of bacteria enhances the release of immunomodulatory bacterial products. Several studies have highlighted the anti-inflammatory potential of LysM using the LysM^-/-^ model. Indeed, subcutaneous injection of PG or lysozyme-sensitive bacteria *Micrococcus luteus* led to poor bacterial digestion and increased immune response and tissue injury in LysM^-/-^ mice, with increased immune infiltration (56), regardless of the compensatory expression of lysozyme P in Paneth cells and in macrophages (56). However, higher levels of IL-10 were measured in the lungs of LysM^-/-^ mice upon *Klebsiella pneumoniae* infection, despite increased susceptibility to infection as compared to WT mice (57). On the other hand, in the same study, mice overexpressing lysozyme M displayed an increased survival rate upon infection, highlighting the protective effect of lysozyme M. Additionally, supplementation with hen egg lysozyme (HEL) was shown to reduce colitis symptoms and pro-inflammatory cytokine production in a porcine model of intestinal inflammation (44). Due to the dual pro- and anti-inflammatory roles of lysozyme in immune response, a model of the regulated function of LysM has been proposed where it could prevent dysbiosis and act as an immunomodulator (33).

Lysozyme has also been described as an anti-cancer agent. For instance, TGF-β and FoxP3 levels were increased upon HEL treatment, suggesting the induction of regulatory mechanisms (44). The increased suppressive capacity of peritoneal Lyz2^+^ myeloid cells after Nod2 stimulation needs further investigation to decipher if the mechanism might involve migration or survival of these lysozyme-expressing cells. Alternatively, it remains possible that compensatory mechanisms may occur within the large intestine of NOD2^▵Lyz2^ mice to prevent the conversion of extravasating Ly6C^hi^ monocytes into phenotypically and functionally distinct cells within the inflamed colonic lamina propria (58). Further research is also needed to investigate if NOD2 expression in phagocytes may favor colorectal cancer through limiting lysozyme-dependent anti-cancer properties such as direct activation of immune effectors and immunosuppressive antioxidant properties (52).

An additional issue was related to the exact nature of the cells expressing lysozyme M that remains to be defined. It is unclear which cells that secrete lysozyme M would retain some inflammatory properties upon activation of Nod2 signaling. Indeed, the use of the yellow color gene (YFP) tracing model revealed that the *Lyz2* gene is expressed in discrete populations of myeloid cells (59). Specifically, subsets of neutrophils, monocytes, macrophages, type 2 conventional dendritic cells (DC2), and plasmacytoid dendritic cells (pDC) can express at varying degrees the Lyz2-encoding gene (38, 59, 60). Only part of the answer to this puzzle may lie in other cells than cDCs as colitis severity and tumor burden were similar between control and *Clec9^Cre/+^;Nod2^fl/fl^* littermates (data not shown). This said, the loss of Nod2 expression in neutrophils may probably improve tissue repair and tumor progression through modulation of the Th17-mediated immunity, as observed in *LysM^Cre^*;*Mcl1^fl/fl^* mice (61). Alternatively, one may also postulate that the lowered disease severity of *LysM^Cre/+^;Nod2^fl/fl^* mice may result from the ability of Nod2 to convert Ly6C^high^ into Ly6C^low^ monocytes (38) and to skew monocyte fate decision (20). Accordingly, Nod2 is known to interact with the interferon-regulatory factor 5 (Irf5) that promotes Ly6C^hi^ monocyte trafficking to the peritoneal cavity (62) and defines the commitment to the M1 macrophage polarization (63). Further research is needed to assess whether Nod2 signaling may guide the pathogenic properties of a specific subset of pro-tumorigenic macrophages that may influence the tissue repair process and carcinogenesis (64). However, it is worth noting that we failed to observe similar changes in the myeloid content of the lamina propria of *LysM^Cre/+^;Nod2^fl/fl^* mice when compared to *LysM^Cre/+^*;*Irf5^fl/fl^* mice (65) even though we did not use single-cell RNA sequencing approaches. Collectively, our work suggests that loss of NOD2 in lysozyme-expressing phagocytes aids in the resolution of inflammation. Such compensatory mechanisms in the colon could be targeted to promote anti-inflammatory properties and immune surveillance against colorectal tumors.

## Materials and methods

### Mice

All animal studies were approved by the local investigational review board of the Institut Pasteur of Lille (N°28010-2016012820187595). Animal experiments were performed in an accredited establishment (N° B59-108) according to governmental guidelines N°86/609/CEE. Age-matched and gender-matched C57BL/6J WT, Nod2-deficient mice (Nod2^-/-^), Nod2^ΔLyz2^, Nod2^fl/fl^ have free access to standard laboratory chow diet in a temperature-controlled SPF environment and a half-daylight cycle exposure. C57BL/6J WT mice were purchased from Janvier Laboratories, France. Ly5.1 WT mice (CD45.1) were purchased from Charles Rivers Laboratories, France. *Nod2^–/–^* mice were provided by R.A. Flavell (Yale University School of Medicine,

Howard Hughes Medical Institute). We thank Dr Philip Rosenstiel (Institute of Clinical Molecular Biology, Kiel, Germany) for providing the Nod2^fl/fl^ mice, and the Jackson (stock #004781) for the LyzM-Cre mice (also known as Lyz2-Cre) (21). Conditional knockout of Nod2 in the lysozyme M expressing cells was established by crossing *LyzM-*Cre with *Nod2* ^fl/fl^ mice, resulting in *Nod2* ^ΔLyz2^ or *Nod2* ^fl^.

### Induction of relapsing-remitting colitis and inflammation-driven colon carcinogenesis

Relapsing-remitting colitis was induced by giving mice 2% (wt/vol) DSS (TdB Consultancy) for a period of 5 days followed by normal drinking water for 2 days or 7 days when mentioned. DSS was dissolved in drinking water and changed every 3 days. Signs of morbidity, including body weight, stool consistency, occult blood, or the presence of macroscopic rectal bleeding, were checked daily. At specific time points throughout the course of the challenge, mice were autopsied to assess the severity of the disease by measurement of colon lengths and cell composition by flow cytometry. To induce colorectal tumorigenesis, mice were challenged intraperitoneally with AOM (8 mg/kg body weight; Wako; Sigma-Aldrich) 5 days before four cycles that consist of a 5-day period of 2% DSS treatment (wt/vol) followed with a week of regular water, as previously described (Neufert C, 2007). The distal colon was dissected out and cut transversely. Tumor numbers were assessed, and, in some experiments, the colon was stained with 5% methylene blue solution for a few seconds to better visualize small colon polyps. Tissue specimens were collected and kept frozen until further quantification of transcript levels. Mouse endoscopy was performed using the Coloview high-resolution system (Karl-Storz) to assess the presence of ulcerations and adenomas within the colon.

### Isolation of mouse colonic lamina propria cells

Lamina propria Mononuclear Cells (LPMC) were prepared from murine intestines by enzymatic digestion as previously described (66). Briefly, cells were isolated from colons, after removal of epithelial cells, by enzymatic digestion with 200mg/ml fungizone, 1.25 mg/ml collagenase D (Roche Diagnostics), 0.85 mg/ml collagenase V (Sigma-Aldrich), 1 mg/ml dispase (Life Technologies), and 30 U/ml DNaseI (Roche Diagnostics) in complete RPMI 1640 for 30–40 min in a shaking incubator until complete digestion of the tissue. After isolation, cells were passed through a 40μm cell strainer before use (BD biosciences).

### Generation of bone marrow-derived macrophages

Bone marrow cells were flushed out of the mouse bones with complete RPMI 1640 (Gibco). A single-cell suspension was then prepared by repeated pipetting. Bone marrow-derived macrophages (BMDMs) were generated for 7 days in IMDM, supplemented with L-glutamine, MEM non-essential amino acids, sodium pyruvate, penicillin/streptomycin (all from Thermo Fisher), and 10% heat-inactivated fetal calf serum (GE Healthcare). The medium was supplemented with 20% supernatant of L929 cells (M-CSF-producing cells).

### Cytokine measurement

Cytokine levels were determined by ELISA kits (DuoSet), according to protocols provided by R&D Systems.

### Flow cytometry

Single-cell suspensions were stained and analysed using a FACS LSRFortessa™ system (BD Biosciences). Dead cells were excluded with the LIVE/DEAD Fixable Violet Dead Cell staining kit (Life Technologies). The cells were then incubated for 10 minutes with purified rat anti-mouse CD16/CD32 (Biolegend, clone 93) and normal mouse serum (Interchim) before being stained with various monoclonal antibodies for 20 minutes in the dark on ice. For mouse cells, lineage-positive cells were excluded using the PerCP5.5-conjugated anti-CD3 (17A2), anti-NK1.1 (PK136), anti-CD19 (6D5), anti-Ly6G (1A8) (Biolegend). PerCP-conjugated anti-CCR3 (83103) added to the lineage staining to exclude eosinophils was from R&D. Alexa Fluor 700-conjugated anti-Ly6C (AL21) was from BD Pharmingen. PECF594-conjugated anti-CD11c (HL3) was from BD Horizon. Allophycocyanin-Cy7-conjugated anti-CD11b (M1/70), PE-Cy7 anti-CD8 (53-6.7), Brilliant violet 510-conjugated anti-MHC Class II (I-A/I-E) (M5/114.15.2), Brilliant violet 650-conjugated anti-CD45.2 (104), were all from Biolegend. PE-conjugated anti-CCR2 (475301) was from R&D systems. The data were analysed with Flowjo software V10.1 (TreeStar).

### THP-1 cell culture and stimulation

The Nod2^+/+^ and Nod2^-/-^ THP-1 monocytic cell line was cultured in RPMI 1640 medium (Gibco) supplemented with 10% heat-inactivated FBS (Gibco), L-glutamine (Thermo Fisher), MEM non-essential amino acids (Thermo Fisher), sodium pyruvate (Thermo Fisher), HEPES (Thermo Fisher), and 0.05mM of 2-mercaptoethanol (Thermo Fisher). Cells were kept in culture at cell concentrations ranging from 2x10^5^ cells/mL to 8x10^5^ cells/mL and routinely verified negative for mycoplasma contamination by PCR analysis. THP-1 macrophages (PMA-Mac) were generated by adding PMA (5ng/ml) for 48h, followed by at least 2 days without PMA (35, 67). PMA-Mac stimulation was performed in 96-well flat bottom plates at 1x10^5^ cells per well in a final volume of 200 μl. Cells were stimulated with two sequential treatments of 24 hours each. For the first 24 hours of treatment, cells were cultured in RPMI complete medium or with LPS at 1ug/mL, and washed to remove the stimuli. The second 24-hour treatment consisted of MDP at 10ug/mL or RPMI medium. Culture supernatants were collected after the second treatment and TNF-α levels were quantified by ELISA using the Human TNF-alpha DuoSet ELISA (R&D systems) following manufacturer recommendations.

### Gene expression

RNAs were extracted using the RNEasy mini kit (Qiagen). Isolated RNA was reverse-transcribed with the cDNA synthesis kit (Agilent Technologies), according to the manufacturer’s instructions. The resulting cDNA (equivalent to 500ng of total RNA) was amplified using the SYBR Green real-time PCR kit and detected on a Stratagene Mx3005 P (Agilent Technologies). qRT-PCR was conducted using forward and reverse primers (sequences available upon request). The relative abundance of gene expression was assessed using the 2−ΔΔCt method. Actb was used as an internal reference gene in order to normalize the transcript levels.

### Bone marrow transplantation experiments

Recipient mice underwent a lethal total-body irradiation (2X 5.5Gy, 4h between each dose). Twenty-four hours post-irradiation, mice received 2 × 10^6^ fresh BM cells intravenously. Nod2-deficient animals were irradiated and reconstituted in a 1:1 ratio with BM cells from WT (CD45.1) and Nod2-deficient mice (CD45.2). Blood was collected in heparin-containing tubes 7–8 weeks after BM transplantation and reconstitution efficiency was checked by flow cytometry (68). DSS was administered for 5 days in the drinking water of mixed-BM chimera mice to induce acute colitis.

### Isolation of myeloid peritoneal cells and suppression assay

Myeloid cells were enriched by negative selection to remove CD3-, CD45-, Ly6G-, and CD49b-expressing cells (Monocyte Isolation Kit, Miltenyi). The suppression test was realized as described in Mainsonneuve et al. (37). CD8 T cells have been isolated by positive selection (Miltenyi Biotech) and cultivated in anti-CD3, anti-CD28 coated plates. Myeloid cells have been added as a ratio of 10 myeloid cells for 1 T cells for 72 h. Proliferation was measured by cytometry by gating on live CD8^+^ cells.

### Mining of publicly available RNAseq dataset in colorectal cancer

RNASeq transcriptome profiles were collected from the Genomic Data Commons Cancer Data Portal (as recent as the V28 release from February 2021) using R software version 4.0.3 and GenomicDataCommons package. Then, data were log-transformed and normalized using DESeq2 R package before being exported to a single tabular text file. Subsequent analyses and data display were realized using several R packages, differential gene expression analysis was done with the limma R package, and differential pathway enrichment analysis and network plot were done with GSVA and igraph R packages coupled with GeneOntology, KEGG, PANTHER, Reactome, WikiPathways and custom pathways databases. Pathway enrichment analysis was realized using EnrichR and MSigDB-Hallmark.

### Statistics

Data were analyzed using Prism6.0 (GraphPad Software, San Diego, CA). Statistical significance was assessed by a non-parametric Mann-Whitney test or two-way ANOVA for multiple comparisons. Values represent the mean of normalized data ± SEM. *, P<0.05; **, P<0.01; ***, P<0.001; ****, P<0.0001.

## Data availability statement

The original contributions presented in the study are included in the article/**Supplementary Material**. Further inquiries can be directed to the corresponding authors.

## Ethics statement

The studies involving humans were approved by TCGA-associated ethics committee. The studies were conducted in accordance with the local legislation and institutional requirements. Written informed consent for participation was not required from the participants or the participants’ legal guardians/next of kin in accordance with the national legislation and institutional requirements. The animal study was approved by local investigational review board of the Institut Pasteur of Lille (N°28010-2016012820187595). The study was conducted in accordance with the local legislation and institutional requirements.

## Author contributions

Conceptualization: CC, KR, PRé, GD-J, MC, and LP. Methodology: CC, KR, OB, MD, NW, GL, CB, MYS, PRé, PRo, GD-J, MC, and LP. Formal analysis: CC, KR, OB, MD, NW, PRé, GL, GD-J, MC, and LP. Investigation: CC, KR, OB, MD, NW, PRé, GL, GD-J, MC, and LP. Writing – original draft: MC and LP. Writing – review and editing: all authors. Visualization: CC, KR, MC, and LP. Supervision: LP and MC. Funding acquisition: LP and MC. All authors contributed to the article and approved the submitted version.

## Supplementary material

Supplementary Figure 1 Validation of *Nod2* depletion in peritoneal and BMDM macrophages. Relative expression of *Nod2* in splenic CD4+ T cells, peritoneal macrophages (Mac), and M-CSF generated bone-marrow macrophages (BMDM) in Nod2^ΔLyz2^ (*LysM^Cre/+^;Nod2^fl/fl^*) and in littermate control flox animals (*LysM^+/+^;Nod2^fl/fl^*). Bars indicate the mean ± SEM of at least three mice per group.

Supplementary Figure 2 The development of colonic tumors was assessed by colonoscopy in LysM^Cre/+^;Nod2^fl/fl^ mice and control flox mice after induction of colorectal cancer by AOM/DSS.

Supplementary Figure 3
*Ccl2* and *Cxcl1* levels are not affected by the lack of Nod2 in myeloid cells during AOM-DSS-induced CAC. *Ccl2* and *Cxcl1* expression levels were measured by RT-qPCRs on colonic tumoral tissues, as described in **Figure 3**. Bars indicate the mean ± SEM. Statistical significance was assessed by a non-parametric Mann-Whitney test. ns, non-statistically significant.

Supplementary Figure 4 Pretreatment of macrophages with LPS increased the MDP response. Macrophages expressing (blue) or not NOD2 (red), were differentiated with PMA (PMA-Mac) and then treated as described in the material and method section to evaluate the MDP responsiveness of LPS-treated cells. TNF-α production was measured as a read-out by ELISA. Bars indicate the mean ± SEM of at least three biological replicates and data are representative of 3 independent experiments. Statistical significance was assessed by ordinary one-way multiple comparisons. *,P<0.05, **,P<0.01, ***,P<0.001 ****,P<0.0001.

Supplementary Figure 5 NOD2 signaling suppressed T-cell proliferation. CD8 T cells stimulated with anti-CD3/anti-CD28 were incubated in the presence of peritoneal phagocytes from either mice pre-treated *in vivo* for 24h with MDP (Myeloid^MDP^) or control mice (Myeloid) for 72H (ratio 1:10). The percentage of T-cell proliferation was assessed by flow cytometry. Bars indicate the mean ± SEM of at least three biological replicates.

Supplementary Figure 6 Control of lysozyme-expressing CD102^+^ macrophage migration by NOD2. **(A)** Mixed competitive bone marrow (BM) chimeras were generated by reconstituting Nod2-deficient recipients with WT (CD45.1; blue) and Nod2^-/-^ (CD45.2; red) BM (ratio 1:1). **(B)** The presence of MHC II^-^ CD102^+^ peritoneal macrophages was assessed by flow cytometry in the BM chimera 5 days after DSS treatment. Bars indicate the mean ± SEM. Statistical significance was assessed by the non-parametric Mann-Whitney U test. **P<0.01.
