# Supplementary figures and images for: Loss of NOD2 in macrophages improves colitis and tumorigenesis in a lysozyme-dependent manner
Source: Front Immunol. 2023 Oct 9;14:1252979. doi: 10.3389/fimmu.2023.1252979 (PMC10590911; doi:10.3389/fimmu.2023.1252979)

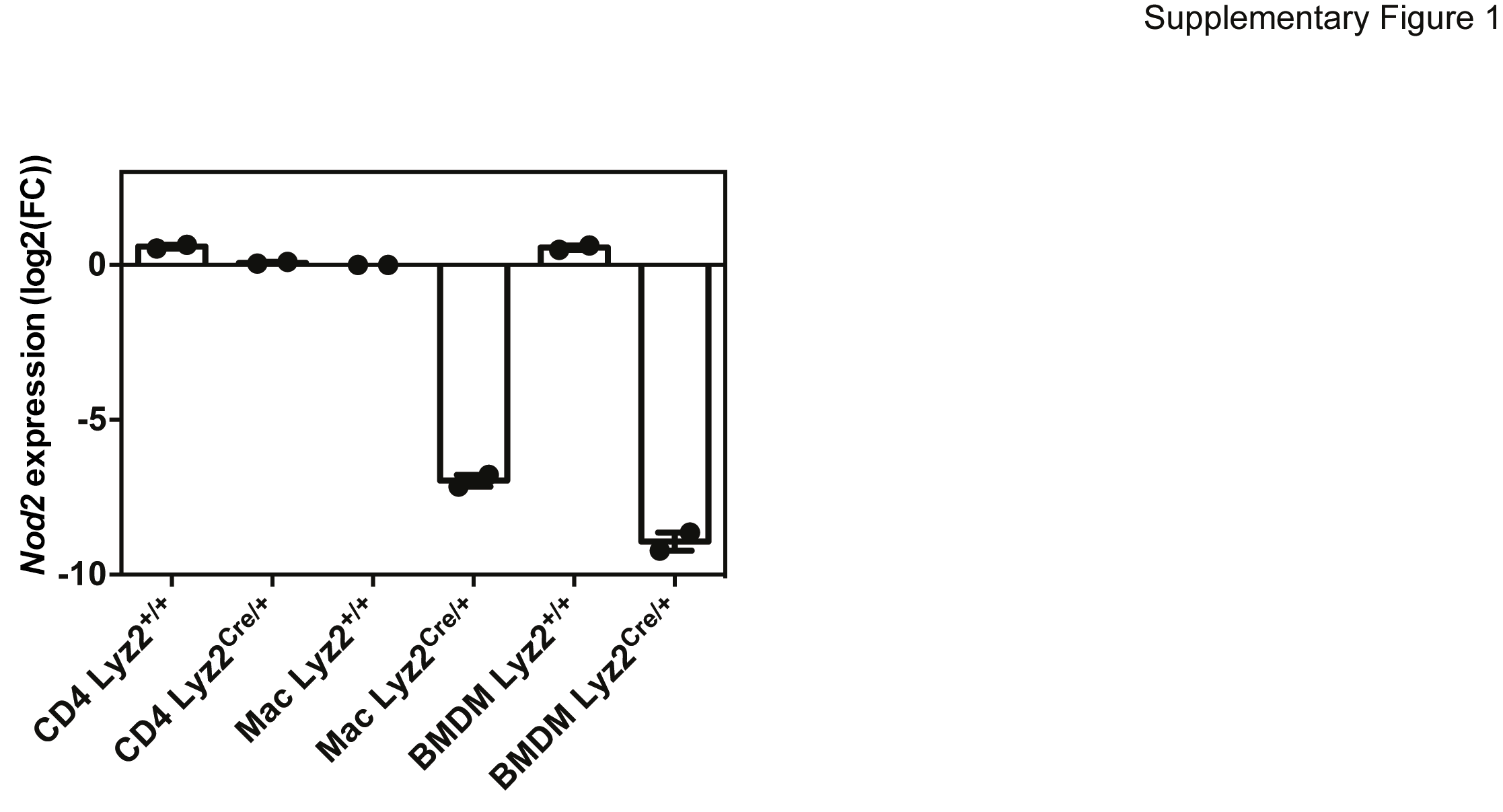

Supplement: Supplementary Figure 1 — Validation of Nod2 depletion in peritoneal and BMDM macrophages. Relative expression of Nod2 in splenic CD4+ T cells, peritoneal macrophages (Mac), and M-CSF generated bone-marrow macrophages (BMDM) in Nod2ΔLyz2 (LysMCre/+;Nod2fl/fl ) and in littermate control flox animals (LysM+/+;Nod2fl/fl ). Bars indicate the mean ± SEM of at least three mice per group. [file Image_1.png]

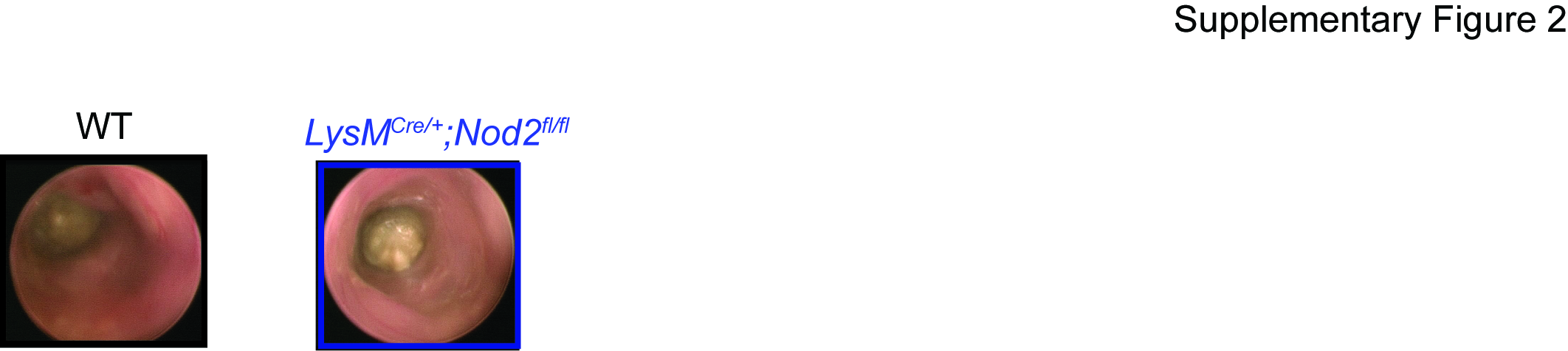

Supplement: Supplementary Figure 2 — The development of colonic tumors was assessed by colonoscopy in LysMCre/+;Nod2fl/fl mice and control flox mice after induction of colorectal cancer by AOM/DSS. [file Image_2.tif]

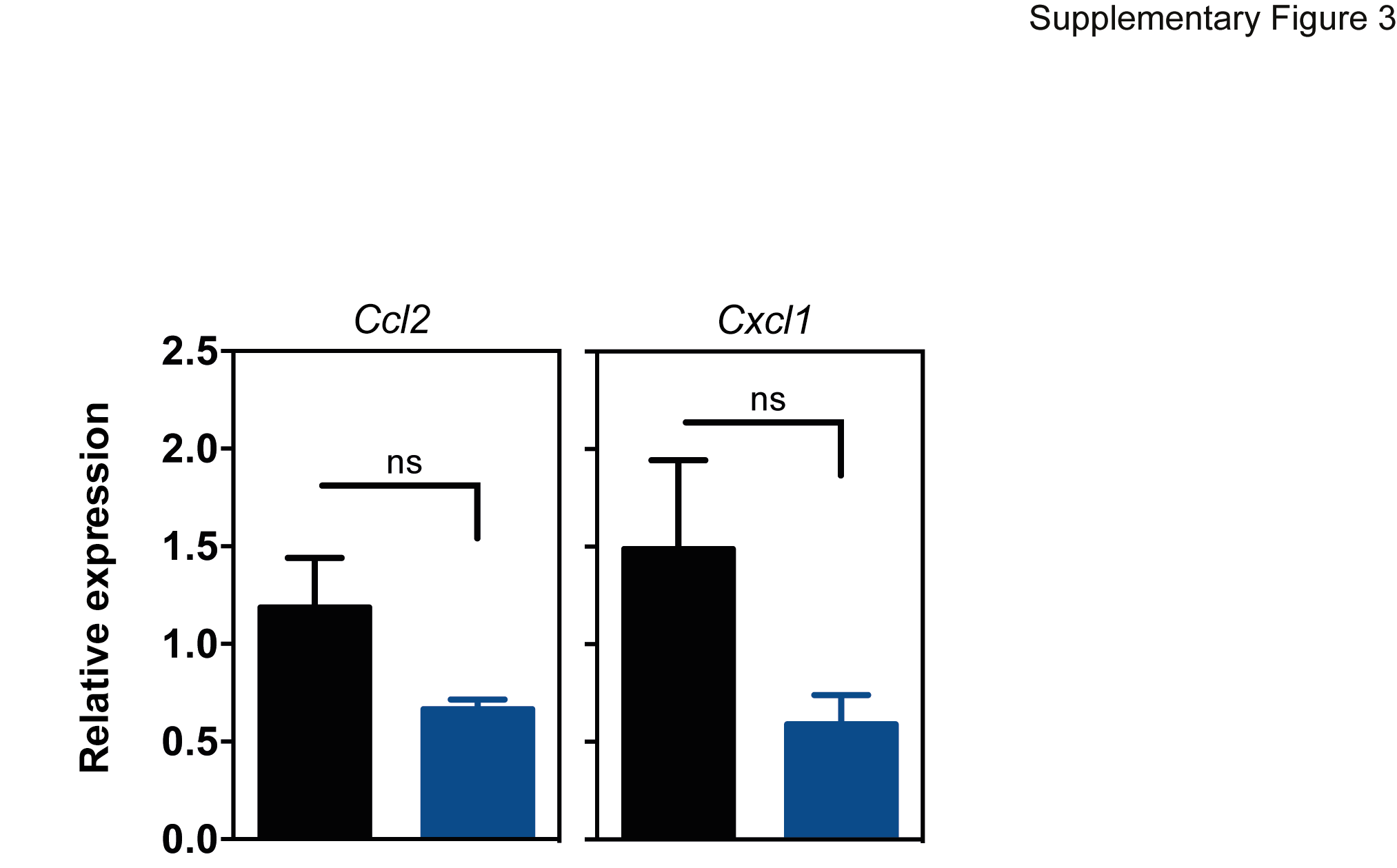

Supplement: Supplementary Figure 3 — Ccl2 and Cxcl1 levels are not affected by the lack of Nod2 in myeloid cells during AOM-DSS-induced CAC. Ccl2 and Cxcl1 expression levels were measured by RT-qPCRs on colonic tumoral tissues, as described in Figure 3 . Bars indicate the mean ± SEM. Statistical significance was assessed by a non-parametric Mann-Whitney test. ns, non-statistically significant. [file Image_3.png]

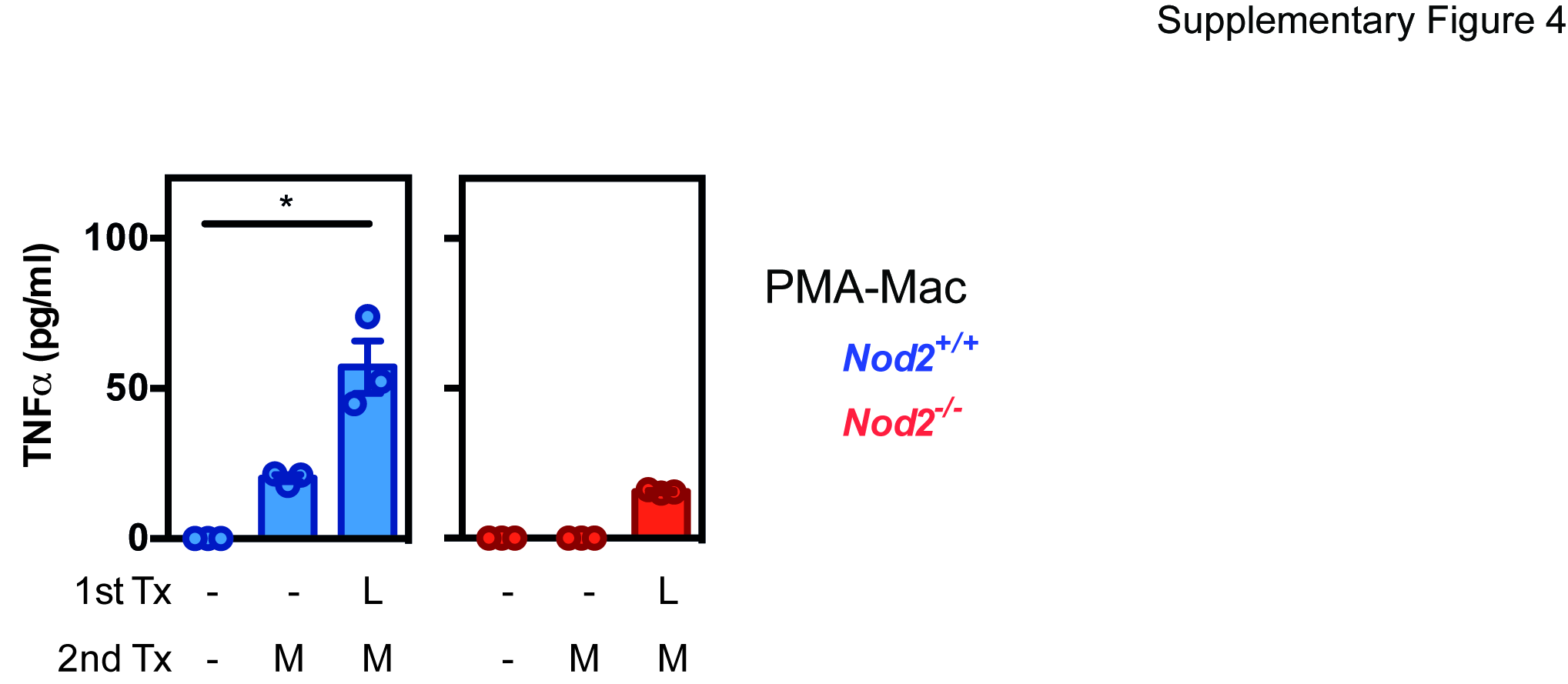

Supplement: Supplementary Figure 4 — Pretreatment of macrophages with LPS increased the MDP response. Macrophages expressing (blue) or not NOD2 (red), were differentiated with PMA (PMA-Mac) and then treated as described in the material and method section to evaluate the MDP responsiveness of LPS-treated cells. TNF-α production was measured as a read-out by ELISA. Bars indicate the mean ± SEM of at least three biological replicates and data are representative of 3 independent experiments. Statistical significance was assessed by ordinary one-way multiple comparisons. *,P<0.05, **,P<0.01, ***,P<0.001 ****,P<0.0001. [file Image_4.tif]

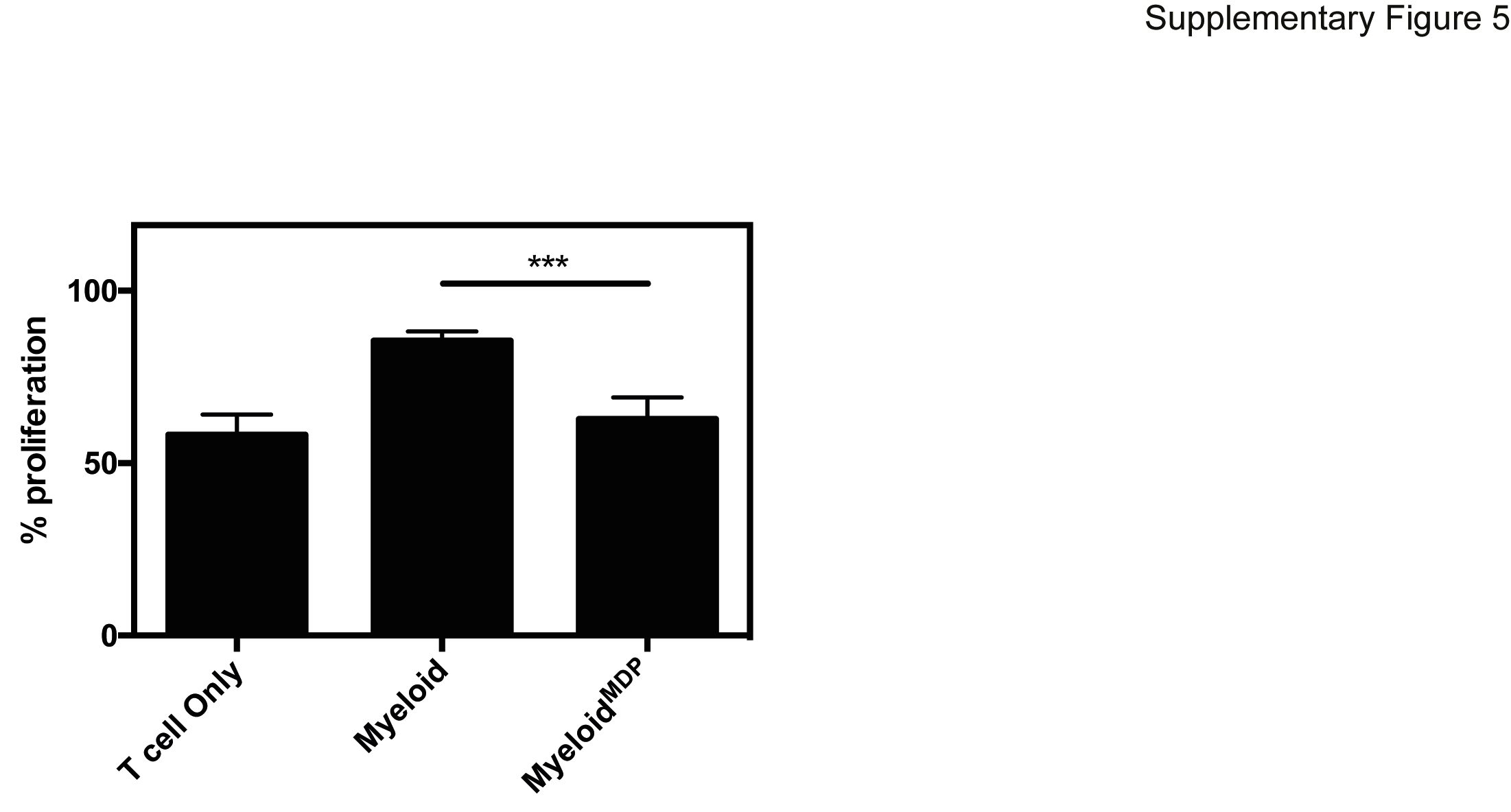

Supplement: Supplementary Figure 5 — NOD2 signaling suppressed T-cell proliferation. CD8 T cells stimulated with anti-CD3/anti-CD28 were incubated in the presence of peritoneal phagocytes from either mice pre-treated in vivo for 24h with MDP (MyeloidMDP) or control mice (Myeloid) for 72H (ratio 1:10). The percentage of T-cell proliferation was assessed by flow cytometry. Bars indicate the mean ± SEM of at least three biological replicates. [file Image_5.png]

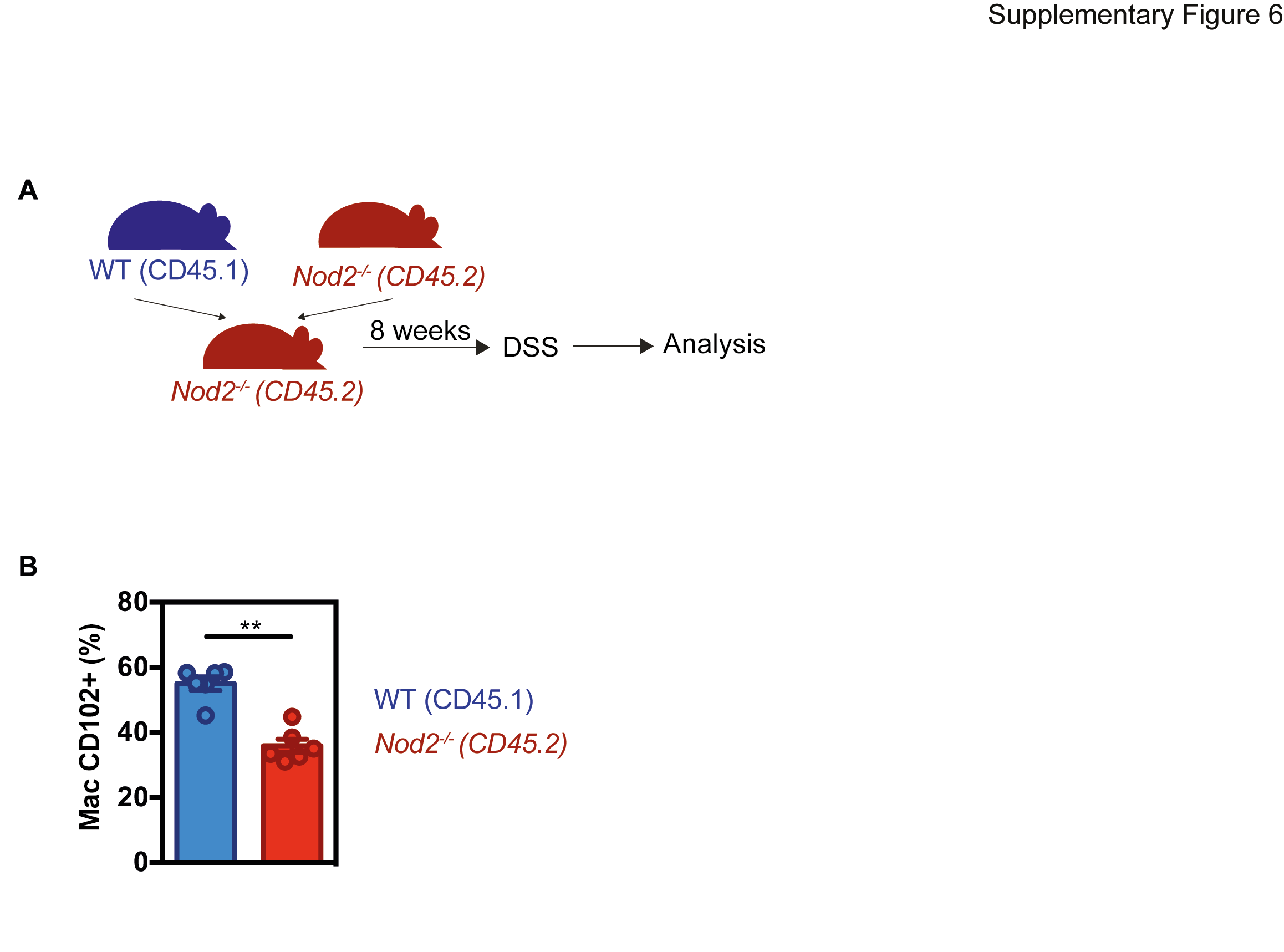

Supplement: Supplementary Figure 6 — Control of lysozyme-expressing CD102+ macrophage migration by NOD2. (A) Mixed competitive bone marrow (BM) chimeras were generated by reconstituting Nod2-deficient recipients with WT (CD45.1; blue) and Nod2-/- (CD45.2; red) BM (ratio 1:1). (B) The presence of MHC II- CD102+ peritoneal macrophages was assessed by flow cytometry in the BM chimera 5 days after DSS treatment. Bars indicate the mean ± SEM. Statistical significance was assessed by the non-parametric Mann-Whitney U test. **P<0.01. [file Image_6.png]
